# Molecular evidence of *Chlamydia pecorum* and arthropod-associated Chlamydiae in an expanded range of marsupials

**DOI:** 10.1038/s41598-017-13164-y

**Published:** 2017-10-09

**Authors:** Delaney Burnard, Wilhelmina M. Huston, Jonathan K. Webb, Martina Jelocnik, Andrea Reiss, Amber Gillett, Sean Fitzgibbon, Scott Carver, Janine Carrucan, Cheyne Flanagan, Peter Timms, Adam Polkinghorne

**Affiliations:** 1 Centre for Animal Health Innovation, Faculty of Science, Health, Education and Engineering, University of the Sunshine Coast, Sippy Downs, 4556 Australia; 2 School of Life Sciences, University of Technology Sydney, Ultimo, Sydney, 2007 Australia; 3 Conservation Medicine Program, Murdoch University, Perth, 6150 Australia; 4 Australia Zoo Wildlife Hospital, Steve Irwin Way, Beerwah, 4519 Australia; 5 School of Agriculture and Food Sciences, University of Queensland, Brisbane, 4072 Australia; 6 School of Biological Sciences, University of Tasmania, Hobart, 7001 Australia; 7 North Queensland Wildlife Care Inc, 4814 Aitkenvale, Australia; 8 Port Macquarie Koala Hospital, Roto House Historic Site, Cnr Lord Street and Roto Place, Port Macquarie, New South Wales, 2444 Australia

## Abstract

The order *Chlamydiales* are biphasic intracellular bacterial pathogens infecting humans and domesticated animals. Wildlife infections have also been reported, with the most studied example being *Chlamydia pecorum* infections in the koala, an iconic Australian marsupial. In koalas, molecular evidence suggests that spill-over from *C. pecorum* infected livestock imported into Australia may have had a historical or contemporary role. Despite preliminary evidence that other native Australian marsupials also carry *C. pecorum*, their potential as reservoirs of this pathogen and other *Chlamydia*-related bacteria (CRBs) has been understudied. Mucosal epithelial samples collected from over 200 native Australian marsupials of different species and geographic regions across Australia were PCR screened for *Chlamydiales*. Previously described and genetically distinct *C. pecorum* genotypes and a range of 16S rRNA genotypes sharing similarity to different CRBs in the broader *Chlamydiales* order were present. One 16S rRNA *Chlamydiales* genotype recently described in Australian ticks that parasitise native Australian marsupials was also identified. This study provides further evidence that chlamydial infections are widespread in native fauna and that detailed investigations are required to understand the influence these infections have on host species conservation, but also whether infection spill-over plays a role in their epidemiology.

## Introduction

Evidence that infections by obligate intracellular bacterial pathogens in the order *Chlamydiales* are generally prevalent in wildlife is increasing^1^, but their impact on the overall health of populations remains unclear^1,2^. The koala (*Phascolarctos cinereus*), an iconic native Australian marsupial, is the most characterised example of the detrimental effects that chlamydial infections can have on the health of individuals and populations^2^. Although asymptomatic infections are most common, *C. pecorum* infections in koalas can result in severe ocular, urinary tract and reproductive disease which, if left untreated, may progress to blindness, incontinence and infertility, respectively. The prevalence of koala *C. pecorum* infections varies from 10–90% in mainland populations, significantly contributing to the rapid decline of this threatened species in certain states of Australia^2,3^. Koalas also carry unique *Chlamydia*-related bacteria (CRBs), although the role these organisms play in disease is yet to be defined^4^.

The origin of *C. pecorum* infection in koalas is currently unclear. It is well known that domesticated livestock, such as sheep and cattle, are susceptible to *C. pecorum* infections. In Australia, genetically similar strains of livestock *C. pecorum* have been identified in koalas, in addition to koala-specific strains^5–9^. This body of molecular evidence has led to the hypothesis that at least some *C. pecorum* infections in koalas may be the result of historical or contemporary pathogen spill-over from livestock sources, consistent with the significant deleterious impact that these infections have on some koala populations^2^. Evidence to support this hypothesis is incomplete, however^1^.

Research into chlamydial prevalence across Australia’s native marsupial species and populations has been severely limited by geography, sample size and the logistical difficulties in sampling. Several marsupial species have been reported to carry *Chlamydiaceae* like *C. pecorum*, as well as both novel and previously described CRBs. *C. pecorum* has been reported in the greater glider, mountain brushtail possum and western barred bandicoot^10,11^; with *C. pneumoniae* also detected in western barred bandicoots^12^. Although some of these marsupials exhibited signs of chlamydiosis, such as ocular disease, there is little evidence that *C. pecorum* has the same pathogenic effect on these marsupials as it does on koalas. Reports such as these, suggest there may be complex and dynamic origins of *Chlamydiales* in marsupials, further complicating our understanding of this pathogen’s evolution. It is possible that *C. pecorum* is a naturally occurring organism within Australia’s marsupial species and the importation of livestock has selectively exacerbated its impact on koala populations^1^. More recently, preliminary investigations of tick species feeding on koalas and other Australian marsupials highlighted a diversity of novel CRBs, raising questions over the presence and impact of these novel bacteria on the marsupial host parasitised as well^13^.

To gain further insight into the prevalence and impact of chlamydial infections on Australian marsupial fauna, we conducted a broad-range *Chlamydiales* order-specific molecular survey. Chlamydial diversity was sampled from non-koala marsupials across a range of geographic regions in Australia alongside a small subset of wild koalas for comparison.

## Results

### *Chlamydiales* PCR positivity in Australian marsupials

Pan-*Chlamydiales* 16S rRNA gene PCR screening of non-koala marsupial swabs from all regions sampled revealed 196/401 positive samples for *Chlamydiales* DNA, translating into a *Chlamydiales* positive rate of 48% for individual animals at one or more anatomical sites (111/231 animals; Table 1). A concurrent screening of 37 koalas tested (57 swab samples) from the East Coast revealed 100% PCR positivity for *Chlamydiales* DNA.

**Table1:** Overview of marsupial species, number and location included in this study and their corresponding individual and tissue site PCR positivity.

Marsupial Species	# of *Chlamydiales* PCR Positive Individuals	Total # of Individuals	PCR positive sites			
			Ocular	UGT*	Rectal	Mixed^#^
***Northern Territory***						
Northern quoll (*Dasyurus hallucatus*)	21	103	5/62	9/59	2/3	9/41
Northern brown bandicoot (*Isoodon macrourus*)	13	37	n/a	n/a	n/a	13/37
Common brushtail possum (*Trichosurus vulpecula*)	7	13	n/a	n/a	n/a	7/13
Fawn antechinus (*Antechinus bellus*)	0	1	0/1	n/a	0/1	n/a
*Total number of marsupial species* = *4*	**41**	**154**	**5/63**	**9/59**	**2/4**	**29/91**
***East Coast***						
Ring tailed possum (*Pseudocheirus peregrinus*)	10	13	4/13	9/13	n/a	n/a
Common brushtail possum (*Trichosurus vulpecula*)	24	26	22/28	20/25	n/a	n/a
Short eared possum (*Trichosurus caninus*)	3	3	3/3	3/3	n/a	n/a
Spotted tail quoll (*Dasyurus maculatus*)	5	5	7/10	6/6	1/1	n/a
Eastern grey kangaroo (*Macropus giganteus*)	3	4	1/4	3/4	n/a	n/a
Swamp wallaby (*Macropus rufogriseus*)	1	1	1/1	1/1	n/a	n/a
Long nosed bandicoot (*Perameles nasuta*)	1	1	1/1	1/1	n/a	n/a
Squirrel glider (*Petaurus norfolcensis*)	1	2	1/2	1/2	n/a	n/a
*Total number of marsupial species* = *8*	**48**	**55**	**40/62**	**44/55**	**1/1**	**n/a**
***Tasmania***						
Common brushtail possum (*Trichosurus vulpecula*)	22	22	44/44	22/22	n/a	n/a
*Total number of marsupial species* = *1*	**22**	**22**	**44/44**	**22/22**	**n/a**	**n/a**
***Totals***						
*Overall total number of marsupial species* = *11*	**111**	**231**	**89**	**75**	**3**	**29**

*UGT includes cloaca and penile swabs; ^#^Mixed sites are a pool of DNA derived from ocular, nasal and cloacal swabs.

**Figure 1:**
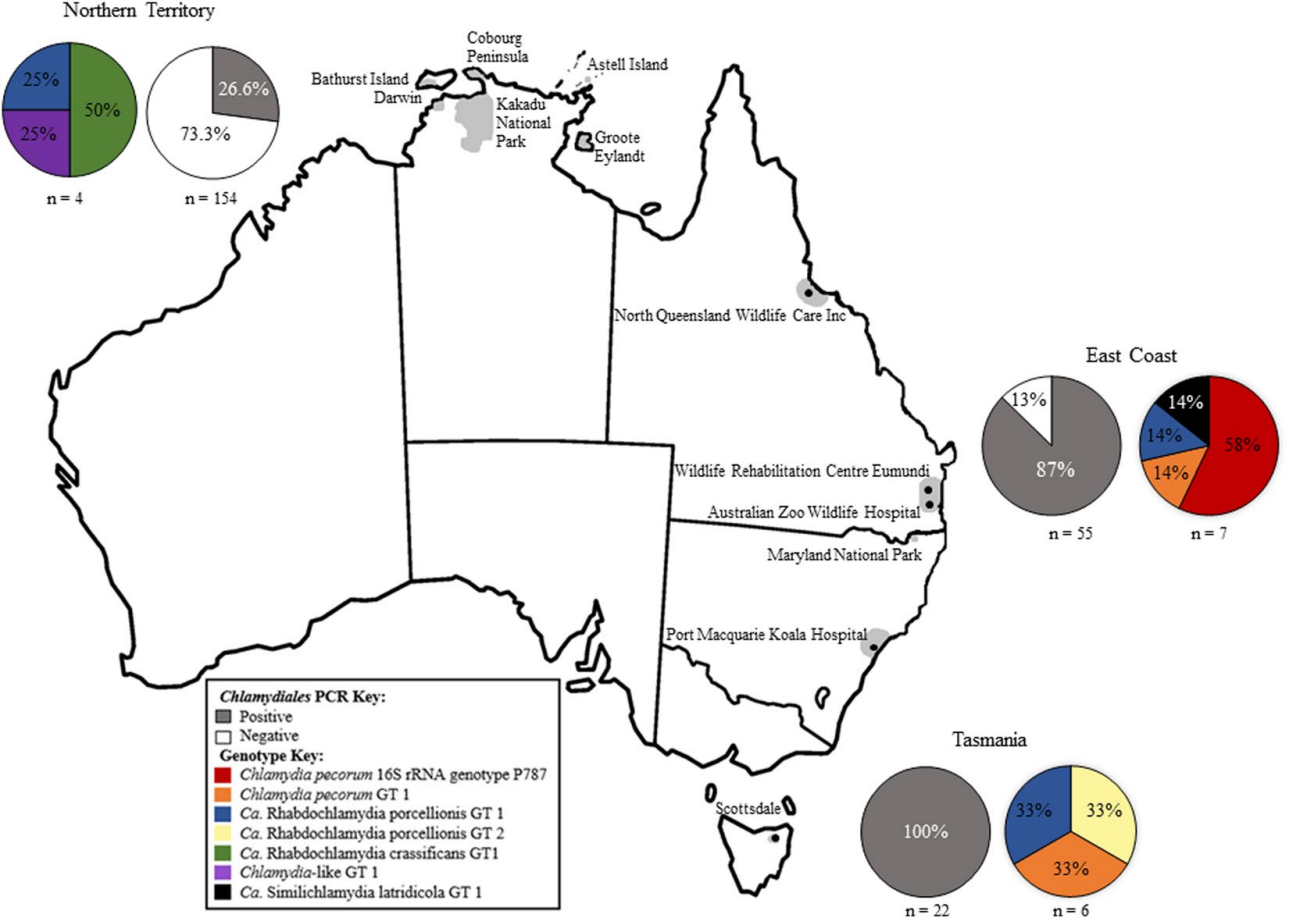
Distribution of *Chlamydiales* genotypes amongst Australian marsupials. Shaded areas represent sampling area for each region. Greyscale pie charts represent number of PCR positive and negative individuals and coloured pie charts represent the abundance and diversity of genotypes detected within each region (see key). Map was modified from (https://commons.wikimedia.org/wiki/File:Australia_states_blank.png) under the Creative Commons Attribution-Share Alike 3.0 Unported license (https://creativecommons.org/licenses/by-sa/3.0/), using Microsoft Windows Version 6.1 Paint and PowerPoint 2013.

To determine the identity of the *Chlamydiales* detected in the marsupials screened in this study, a pan-*Chlamydiales* PCR amplifying an 800 bp fragment of the 16S rRNA gene was targeted. PCR products for 96 non-koala and 43 koala samples were obtained and directly sequenced. Results revealed that 18.7% (18/96) and 58.1% (25/43) of the PCR products for non-koala marsupials and koalas, respectively, returned single sequence results that allowed for direct sequence analysis. The remaining samples resulted in mixed sequences, despite repeated PCR amplification and sequencing efforts, suggesting the majority of samples contained DNA from multiple *Chlamydiales* organisms (data not shown). Nucleotide BLAST (nBLAST) analysis of the single sequence results revealed the presence of one previously described and six novel genotypes belonging to the order *Chlamydiales* ^14,15^ (Table 2, Fig. 1).

**Table 2:** Abundance, identity, SNP differences and non-koala marsupial host information of *Chlamydiales* genotypes identified in this study.

Genotype	Closest BLAST match and % identity (# detected)	# of SNPs/16S rRNA length (bp)	Region, Marsupial and Anatomical Site detected
*Chlamydia pecorum* 16S rRNA genotype P787^*^	*Chlamydia pecorum* CP004035.1 100% (5)	0/744	East Coast, common brushtail possum, ocular; East Coast, common brushtail possum, ocular; East Coast, common brushtail possum, ocular; East Coast, spotted tail quoll, ocular; East Coast, squirrel glider, urogenital.
*Chlamydia pecorum* GT1	*Chlamydia pecorum* CP004035.1 99% (3)	3/600	East Coast, common brushtail possum, ocular; Tasmania, common brushtail possum, ocular; Tasmania, common brushtail possum, ocular.
*Ca*. Rhabdochlamydia porcellionis GT1^*^	*Ca*. Rhabdochlamydia porcellionis AY223862.1 98% (4)	16/759	Tasmania, common brushtail possum, ocular; Tasmania, common brushtail possum, ocular; East Coast, short eared possum, ocular; Northern Territory, northern brown bandicoot, mixed.
*Ca*. Rhabdochlamydia porcellionis GT 2	*Ca*. Rhabdochlamydia porcellionis HF933203.1 93% (2)	30/357	Tasmania, common brushtail possum, urogenital; Tasmania, common brushtail possum, ocular.
*Ca*. Rhabdochlamydia crassificans GT1	*Ca*. Rhabdochlamydia crassificans AY928092.1 99% (2)	7/736	Northern Territory, northern quoll, mixed; Northern Territory, northern quoll, urogenital.
*Chlamydia*-like GT1	*Chlamydia pneumoniae* LN847058.1 92% (1)	56/734	Northern Territory, northern quoll, rectal.
*Ca*. Similichlamydia latridicola GT1	*Ca*. Similichlamydia latridicola KC686679.1 99% (1)	2/745	East Coast, spotted tail quoll, ocular.

*Also detected in East Coast koalas.

### *Chlamydiales* prevalence and genotype diversity varied by geography and marsupial host species

Common brushtail possums harboured the most diverse range of *Chlamydiales*, carrying four of the seven genotypes, with the remaining marsupial species carrying two or less genotypes. Chlamydial prevalence and diversity within non-koala marsupials was also noticeably larger at conjunctival sites (Table 1). PCR positivity of non-koala marsupials varied geographically, with Tasmania having the highest prevalence of 100%, followed by the East Coast sample collection (87%; 48/55) and the Northern Territory collection (26%; 41/154). In the minority of samples where we were able to resolve sequences, the distribution of 16S rRNA sequences across these different geographic locations varied. Only one genotype (*Ca*. Rhabdochlamydia porcellionis genotype 1) was detected in all three geographical locations (Fig. 1). The highest genotypic diversity was found in the East Coast collection where four of the seven different genotypes could be resolved. Three genotypes could each be resolved in the Tasmanian and Northern Territory cohorts (Tables 1 and 2).

### Detection of *C. pecorum* 16S rRNA sequences in koalas and other marsupials

**Figure 2:**
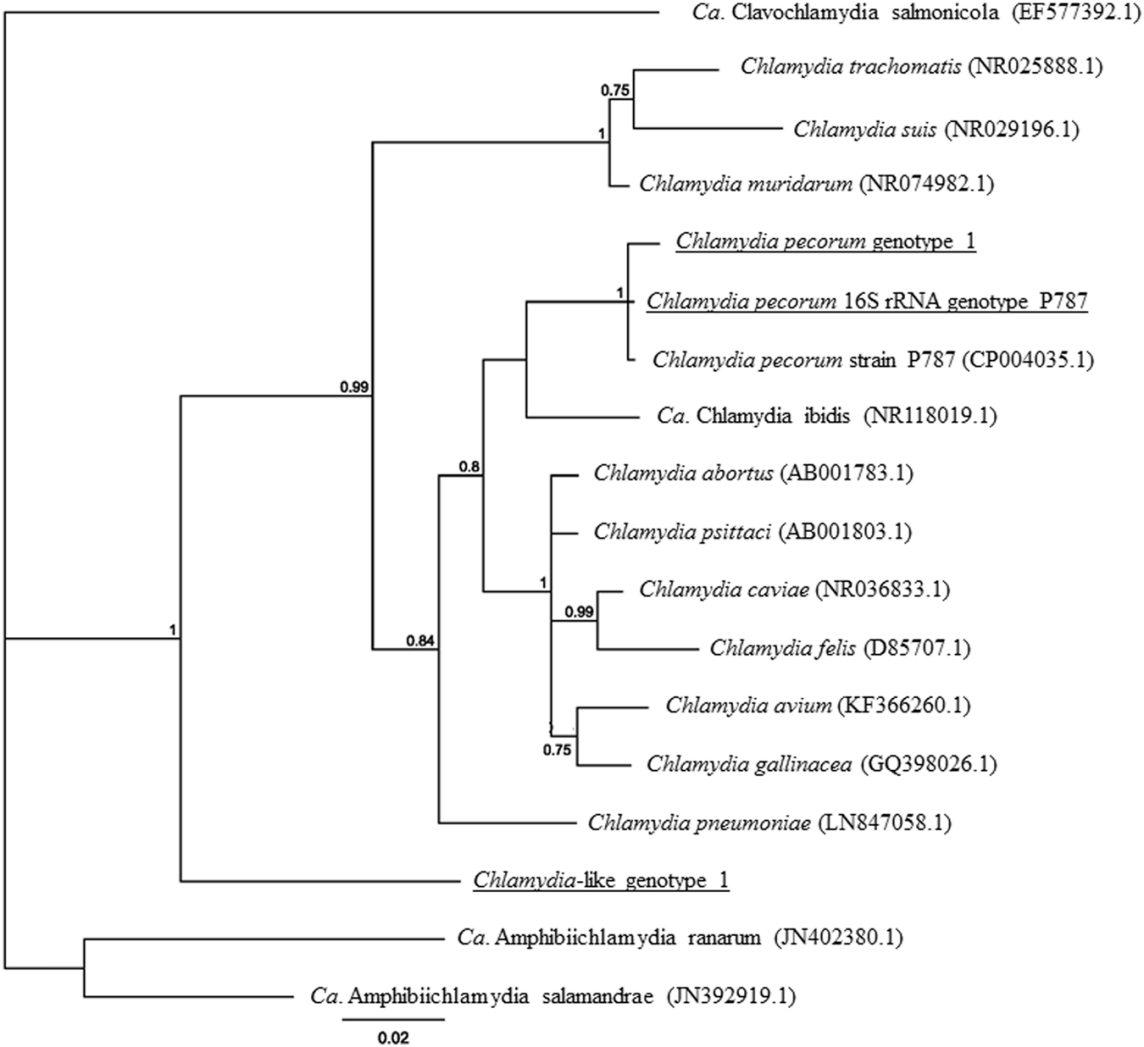
Phylogenetic relationships of *Chlamydiaceae* genotypes identified in Australian marsupials. Bayesian tree incorporating representative 16S rRNA sequences of each species of the genus *Chlamydia* from GenBank, as well as the three partial 16S rRNA genotypes identified in this study. Tree was built using 18 sequences of 588 bp under the HKY85 evolutionary model, posterior probability exceeding 0.75 is shown at internal nodes.

BLAST searching revealed the presence of two distinct *C. pecorum* 16S rRNA sequences in koalas and other marsupials in the East Coast sample set. The first of these partial sequences, detected in three common brushtail possums, one squirrel glider, one spotted tail quoll and 20 koalas was found to share 100% nucleotide identity to previously reported *C. pecorum* 16S rRNA sequences from cattle (P787–CP004035.1; E58–NR_102975.1) and koalas (MC/MarsBar - HQ457465.1; Table 2). For the purpose of this investigation, this 16S rRNA sequence will be referred to as *Chlamydia pecorum* 16S rRNA genotype P787. In addition to this previously described 16S rRNA genotype, a second novel 16S rRNA *C. pecorum* sequence was also detected in three common brushtail possums, two from Tasmania and one from the East Coast (Table 2). This sequence, designated *Chlamydia pecorum* genotype 1, had 99% identity to the top BLAST hit *C. pecorum* strain P787 (CP004035.1) described above, yet differed from this strain by three SNPs. The phylogenetic positioning of these two genetically distinct sequences was confirmed following Bayesian phylogenetic reconstruction (Fig. 2).

### Confirmation of *Chlamydia pecorum* in non-koala marsupials and molecular typing using a *C. pecorum*-specific MLST scheme

**Figure 3:**
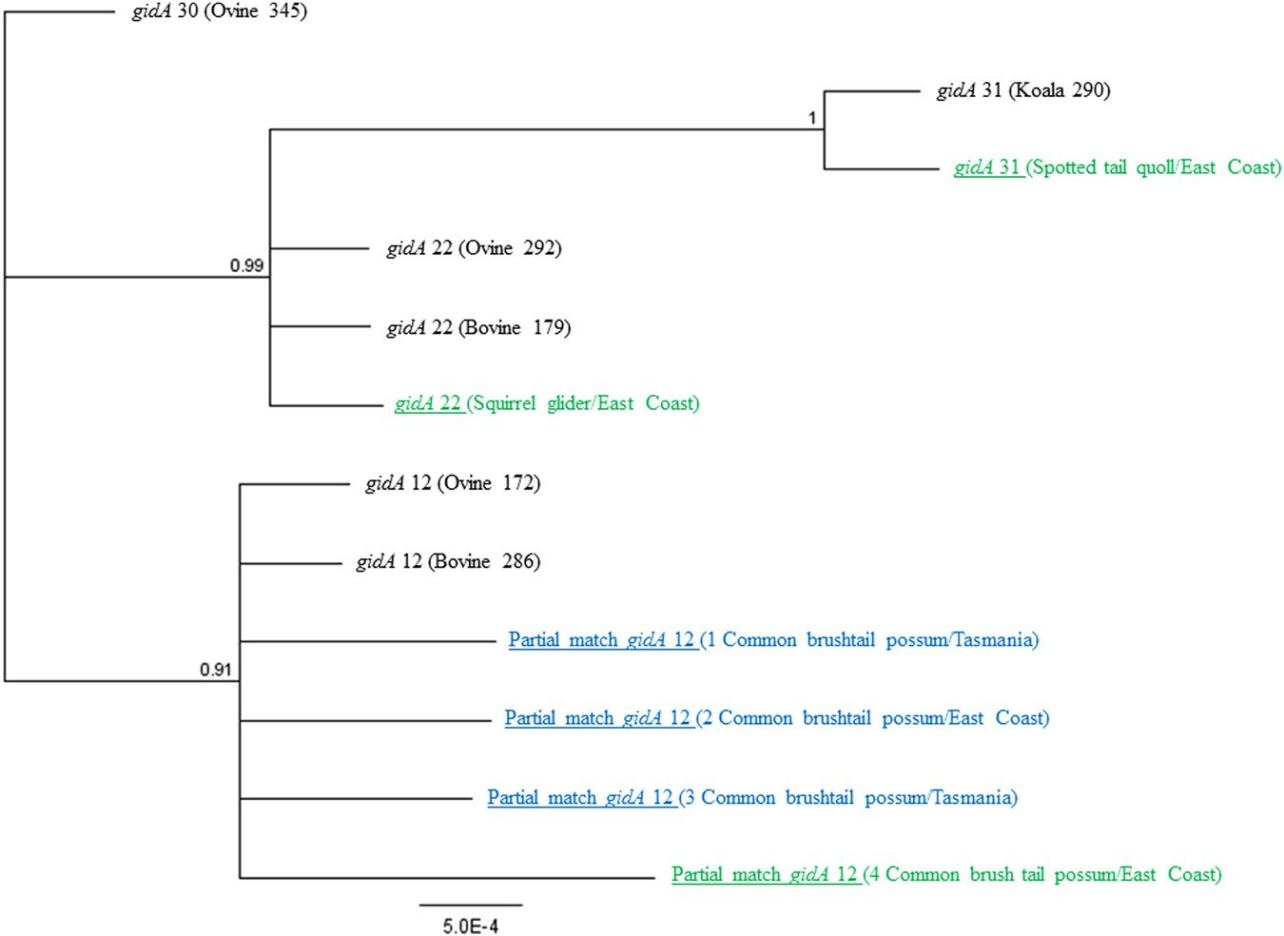
Phylogenetic relationships of *C.  pecorum gidA* alleles identified in Australian marsupials. Bayesian tree incorporating six representative partial *gidA* alleles from PubMLST, as well as the six partial *gidA* alleles identified in this study. Marsupials identified with 16S rRNA as *Chlamydia pecorum* 16S rRNA genotype P787 are coloured green and those identified as *Chlamydia pecorum* genotype 1 are coloured blue. Tree was built using seven sequences of 474 bp under the HKY85 evolutionary model, posterior probability exceeding 0.75 is shown at internal nodes.

In order to confirm the detection of *C. pecorum* in non-koala marsupials and to gain insight into the genetic relationships of these strains to those from other hosts, amplification of seven highly conserved *C. pecorum* MLST housekeeping genes was performed on the eight *C. pecorum* positive non-koala marsupial samples, as previously described^8^. Unfortunately, we were only successful in amplifying one of the seven *C. pecorum* MLST genes (*gidA*) from six of the non-koala marsupial samples. Analysis of these *gidA* gene sequence fragments and comparisons to previously described *gidA* sequences deposited in the *C. pecorum* PubMLST database^16^ revealed a 100% sequence match for the spotted tail quoll sample to *C. pecorum* *gidA* allele 31, an allele previously described in koalas. The squirrel glider sample was an exact match to *C. pecorum* *gidA* allele 22, which has previously been described in both sheep and cattle. However, the four common brushtail possum samples were all identified to have novel *C. pecorum* *gidA* allele 12 sequences, each encompassing one to two unique SNPs (Fig. 3), when compared to *gidA* alleles derived from sheep and cattle.

### Detection of arthropod-associated *Chlamydiales* 16S rRNA sequences in Australian marsupials

**Figure 4:**
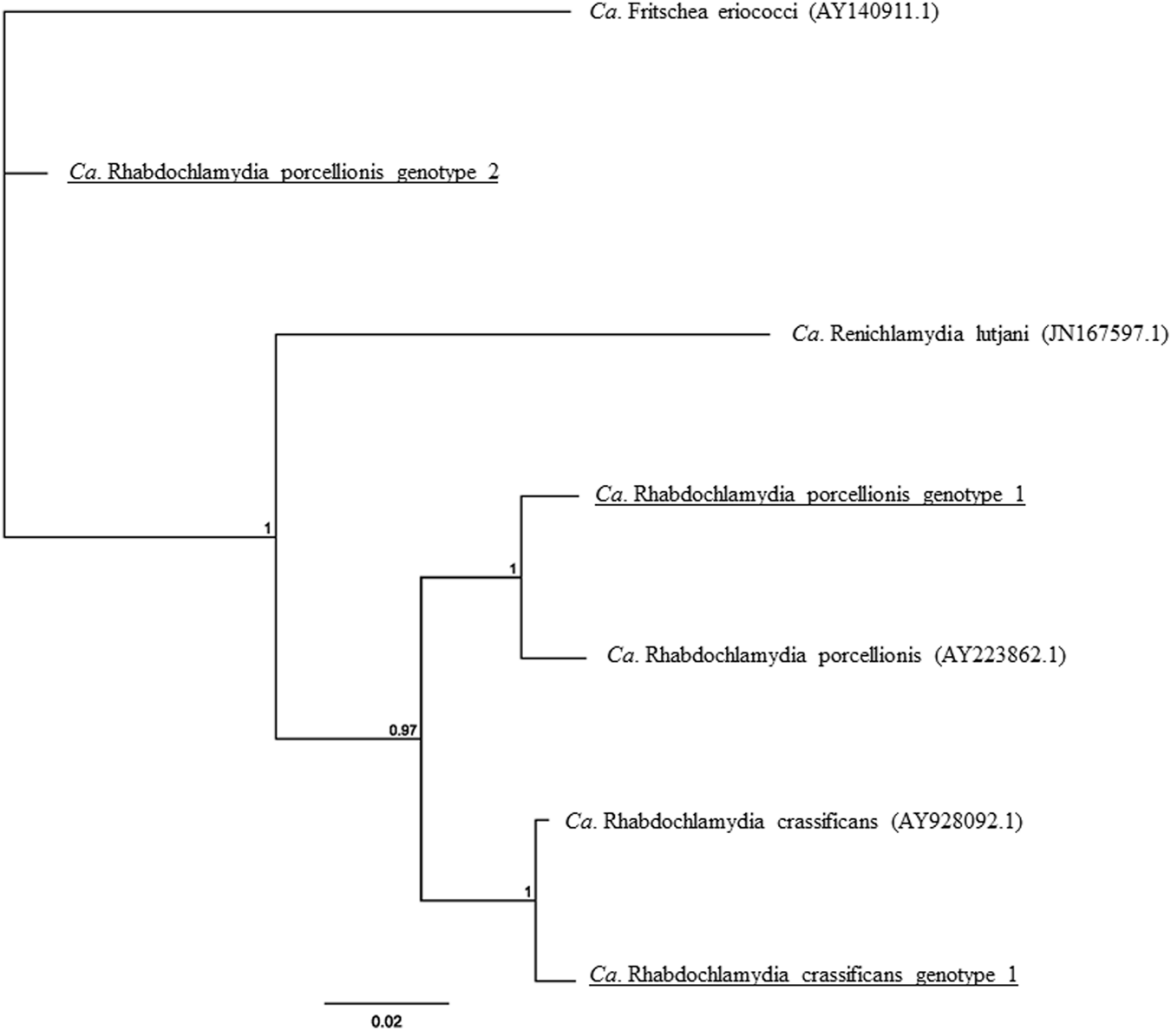
Phylogenetic relationships of *Ca*. Rhabdochlamydiaceae genotypes identified in Australian marsupials. Bayesian tree incorporating representative 16S rRNA sequences of each genus within the *Ca*. Rhabdochlamydiaceae from GenBank, as well as the three partial 16S rDNA genotypes identified in this study. The  tree was built using seven sequences of 368 bp under the HKY85 evolutionary model, posterior probability exceeding 0.75 is shown at internal nodes.

The most geographically distributed CRB genotype (*Ca*. Rhabdochlamydia porcellionis genotype 1) detected in this study was 100% identical to a recently described novel *Ca*. Rhabdochlamydia porcellionis genotype (KX774317) detected in marsupial-feeding tick species removed from koalas^13^. This genotype is previously described as having 98% BLAST similarity to *Ca*. Rhabdochlamydia porcellionis (AY223862.1) and branches as a distinct lineage (Fig. 4). *Ca*. Rhabdochlamydia porcellionis genotype 1 was detected in two common brushtail possums, one short-eared possum and one northern brown bandicoot across the East Coast, Northern Territory and Tasmania (Fig. 1) and, additionally, in five koalas from the East Coast sample collection.

Beyond this previously described arthropod-associated *Chlamydiales* sequence, a novel *Ca*. Rhabdochlamydia crassificans strain was also detected in two northern quolls exclusive to the Northern Territory (Fig. 1). *Ca*. Rhabdochlamydia crassificans genotype 1 exhibited 99% BLAST identity to the closest match *Ca*. Rhabdochlamydia crassificans (AY928092.1), differing by seven SNPs (Fig. 4, Table 2). A sequence potentially representing a novel genus diverging from the previously described *Ca*. Rhabdochlamydiaceae (Fig. 4) was also detected in two common brushtail possums, exclusively to the Tasmanian region. This partial 16S rRNA genotype, *Ca*. Rhabdochlamydia porcellionis genotype 2, demonstrated 93% BLAST identity to *Ca*. Rhabdochlamydia porcellionis (HF933203.1) (Fig. 1, Table 2) as well as previously described novel *Ca*. Rhabdochlamydia genotypes identified in ticks^13^.

### Detection of other CRB 16S rRNA sequences in Australian marsupials

A partial 16S rRNA gene sequence potentially representing a novel genus within the family *Chlamydiaceae* was also detected in a northern quoll from the Northern Territory (Fig. 1). Fine-detailed phylogenetic analysis of this 16S rRNA sequence against other sequences belonging to species in the family *Chlamydiaceae* suggests that this novel sequence sits between the *Chlamydia* and *Ca*. Amphibiichlamydia genera (Fig. 2, Table 2). This genotype, *Chlamydia*-like genotype 1, exhibited 92% identity to *Chlamydia pneumoniae* (LN847058.1) 16S rRNA from a western barred bandicoot, as well as strains detected in koalas (CP001713.1), African clawed frogs (AF139200.1) and other western barred bandicoots (DQ444323.1).

To conclude the descriptions, we also detected an unusual partial 16S rRNA sequence in a sample from a spotted tail quoll from the East Coast, sharing 99% identity and two single nucleotide differences (Fig. 1) to a previously deposited sequence from the novel chlamydial pathogen of fish, *Ca*. Similichlamydia latridicola (KC686679.1).

## Discussion

Koalas, an iconic Australian marsupial, and globally distributed domesticated animals such as sheep and cattle, are well-known hosts of *C. pecorum* ^2,17^. In the current study, a pan-*Chlamydiales* order specific 16S rRNA gene PCR screening strategy provided molecular evidence that a range of other Australian marsupials also carry *C. pecorum*. A distinct range of novel bacteria from the order *Chlamydiales* were also found, some of which have been previously reported in ticks^18,19^, including those found parasitising Australian marsupials^13^.

In this study, *C. pecorum* 16S rRNA genotype P787 and *C. pecorum* genotype 1 accounted for 44.4% of the partial 16S rRNA *Chlamydiales* genotypes obtained, suggesting that *C. pecorum* is more abundant among non-koala marsupials than previously thought. In this study the greatest diversity and abundance of *C. pecorum* was found in common brushtail possums, suggesting that some marsupial host species apart from koalas, may be more susceptible to *C. pecorum* infection than others. Possums have previously been identified as a carrier of *C. pecorum* ^10^. While we need to treat the geographic distribution data with caution since we were only able to resolve sequences for the minority of PCR-positive samples we detected, it is notable that the majority of *C. pecorum* positive samples were detected in the East Coast. This region strongly overlaps with the host range of koalas that are endemically infected with *C. pecorum* and, indeed, the majority of animal hospitals that were used for sampling in this study commonly receive high numbers of koalas into care with chlamydiosis^20,21^. Notably, animals sampled in the Northern Territory where koalas are not found, were free of *C. pecorum*. Inconsistent with this trend was the more surprising detection of* C. pecorum* positive samples from Tasmanian brushtail possums. Wild koalas do not inhabit Tasmania and, to the author’s knowledge, this report represents the first reported detection of chlamydial pathogens in Tasmanian fauna. Tasmania was last connected to mainland Australia during the Pleistocene (~25–8, 000 years ago)^22^ suggesting *C. pecorum* (i) has either been introduced to Tasmania by domestic species such as sheep and cattle, (ii) naturally occurs in possums from this region, and/or (iii) infections in Australian marsupials predated the separation of Tasmania from the mainland.

While only preliminary in nature, a combination of partial 16S rRNA *Chlamydiales* and *C. pecorum* *gidA* gene sequencing suggests that the *C. pecorum* strains detected may be genetically diverse and may differ from those previously detected in koalas and Australian livestock^8,9^. This was most apparent in the *gidA* sequences, where several previously described and novel *gidA* haplotypes were detected, suggesting the possibility of marsupial host specific strains and no favourable association with koalas or livestock based on the *C. pecorum* *gidA* data available to date. Unfortunately, we were not able to gain any further insight into the genetic diversity of these strains since we failed to amplify any of the remaining house-keeping genes utilised in previously described chlamydial MLST schemes (Jelocnik *et al*., 2013). While it is not entirely clear why this was the case, we expect it was due to the presence of only very low levels of *C. pecorum* DNA in these samples, combined with varying sensitivities of each of the individual PCR assays included in this scheme. Further confirmation of strain diversity and insight into the relationships of these strains is not possible without detailed genomics studies to compare these non-koala Australian marsupial *C. pecorum*  strains to those from  koalas, sheep and cattle.

In the absence of such data, a partial  answer regarding the origin and impact of these infections in Australian marsupials may lie in the disease presentation of *Chlamydia* positive animals in this study, as well as the ecological niche that these animals occupy. All 231 individual non-koala marsupials screened in this study were void of any classical signs of chlamydiosis despite several being *C. pecorum* positive at either ocular or urogenital sites. However, the comparison koalas infected with the same strain (based on partial 16S rRNA gene sequence), ranged from being asymptomatic to presenting with severe chlamydiosis (data not shown). Previously, ocular and urogenital chlamydiosis was observed in approximately 20% of *C. pecorum* negative non-koala marsupials and *C. pecorum* positive test results could only be obtained from three quarters of animals showing classical signs of disease^10,11^. It is also interesting to note that in this and the previous study, only arboreal marsupials such as possums, gliders (and koalas) were thus far, found to be more often *C. pecorum* positive than ground-based marsupials, such as bandicoots which are more often *C. pneumoniae* positive^10–12^. For example, in our study, possums sampled from Tasmania had 100% *Chlamydiales* PCR positivity with *C. pecorum* strains identified twice. The quolls and bandicoots, predominantly sampled from the Northern Territory, had 27% *Chlamydiales* PCR positivity and no *C. pecorum* was identified; the *Chlamydia*-like genotype 1 identified from the quoll was, in fact, more similar to *C. pneumoniae*. While clearly too early yet to draw formal conclusions, we speculate that (i) *C. pecorum* infections are primarily asymptomatic in Australian marsupials and that (ii) ecological niche may play a role in predisposing individuals to infection. As to why koalas experience severe chlamydiosis, further comparative genomics analysis of koala and non-koala marsupial *C. pecorum* may reveal undetected *C. pecorum* strain diversity or other pathogenic agents, including other *Chlamydiales* that may predispose koalas to disease in a way that other marsupials are not.

Beyond *C. pecorum*, novel *Chlamydiales* were highly abundant amongst non-koala marsupials and made up approximately 50% of the genotypes retrieved in this screen (Table 2, Fig. 1). “Uncultured *Chlamydiales*” were previously described as the most abundant taxa in non-koala marsupials, even in smaller screens^10,11^, with the impact of these infections on animal health still unknown. Compared to the previous studies, the majority of the novel CRBs identified from marsupials in this study, however, are thought to primarily be limited to infections of arthropods^13,18,23,24^. Indeed, the predominant CRB sequence detected was a partial *Ca*. Rhabdochlamydia porcellionis 16S rRNA gene sequence (Genotype 1) in a range of marsupials that was 100% identical to a genotype we recently described in engorged marsupial feeding tick species *Ixodes tasmani* and *Ixodes holocyclus* removed from koalas^13^. In addition to this genotype being present in four non-koala marsupials distributed across all three geographic regions sampled, five of the 37 koalas we screened were also positive for this partial 16S rRNA gene sequence. Curiously, this genotype was found in marsupials in all three regions included in this study, yet we only found this genotype in ticks removed from koalas in Queensland, a sampling region included in the East Coast. Although the sample sizes were smaller, ticks removed from other marsupial species did not carry this genotype^13^. If this observation is true, this is surprising given that *I. tasmani*, the common marsupial tick is the most widely distributed *Ixodes* species and is found throughout mainland Australian as well as Tasmania. The fact that marsupials and their parasitising arthropods share the same CRBs could implicate ticks as a route of transmission for these novel Chlamydiae. This suggestion was also made by a recent study investigating the occurrence of CRBs in ticks and skin biopsies from suspected tick bite individuals in Finland. Multiple sequences obtained from skin biopsies were found to be most closely related to CRBs detected from ticks species found in Europe, and in some cases, Finnish ticks^25^. Further investigation into the pathogenesis and biology of novel CRBs is obviously warranted, especially given their presence in potential arthropod vectors.

## Conclusions

This body of work further reinforces that chlamydial infections, including the previously described koala pathogen, *C. pecorum*, and other CRBs are widespread in Australian wildlife with some hosts more commonly infected than others. It appears that these infections do not cause traditional chlamydial diseases, certainly not like the debilitating chlamydial diseases that are observed in koalas, although longitudinal health studies would be required to ascertain this further. In the case of *C. pecorum*, what answers this holds about the origin and evolution of this pathogen in Australian marsupials is difficult to assess without further insight into the genetic differences that may exist between marsupial *C. pecorum* genotypes identified in this study with koala, sheep and cattle strains. Interestingly, the identification of a arthropod-associated *Ca*. Rhabdochlamydia porcellionis genotype in various marsupials, including koalas and koala-feeding tick species also suggests that arthropods are not the only host to this organism and further investigation into the pathogenesis and transmission of this and related CRBs is required.

## Materials and Methods

### Sampling

This study utilised a combination of (i) retrospective screening of wildlife samples collected as a part of other investigations and (ii) prospective sampling studies to investigate the prevalence of chlamydial infections in Australian marsupials from different geographic regions across Australia. Samples were obtained by swabbing the mucosal epithelia of conjunctiva, urogenital tract, rectal and penile tissues using Copan rayon tipped, aluminium-shafted applicator swabs (Interpath Services, Heidelberg West, Australia) for prospective sampling and both Copan rayon tipped, aluminium-shafted applicator swabs and Copan FLOQSwabs (Interpath Sevices) for retrospective sampling. Cumulatively, 401 swabs were opportunistically collected from 231 individual non-koala marsupials, comprised of 11 species across four Australian regions. The marsupial species, number of each marsupial and swab site are presented in Table 1. As a sample comparison set, 57 swabs from 37 koalas were opportunistically screened, as well.

All experimental protocols for the sampling of marsupials in this study were approved by the University of the Sunshine Coast Animal Ethics Committee (ANS1539). All methods were carried out in accordance with the 2013 Australian National Health and Medical Research Council ‘Australian code for the care and use of animals for scientific purposes’.

### Retrospective sampling

#### Northern Territory

A total of 217 DNA extracts from 154 individual non-koala marsupials spanning four species were included from the Northern Territory (Table 1). DNA extracts of 91 mixed mucosal epithelial sites (eye, nose, cloaca) from 91 individuals, representing four marsupial species were collected as part of a large-scale study of small mammal health in the Northern Territory Australia^26^. Another 126 DNA extracts of ocular, urogenital and rectal mucosal epithelial sites from 63 individuals, representing two marsupial species were collected as part of a large-scale study of a reintroduction program of the northern quoll.

### Prospective Sampling

#### East Coast

One hundred and eighteen swabs were opportunistically taken from various mucosal epithelial sites (eye/s, urogenital tract, rectum and penis) of 55 non-koala marsupials representing eight marsupial species (Table 1) presenting to collaborating Australian veterinarian and wildlife care centres in New South Wales and Queensland. A total of 57 swabs from 37 individual koalas were taken from eye and urogenital mucosal epithelial sites of 9 and 28 koalas presenting to Wildlife Rehabilitation Centre at Eumundi, Queensland and Port Macquarie Koala Hospital, New South Wales, respectively.

#### Tasmania

Sixty six swabs were collected from the left and right eye and the urogenital tract mucosal epithelium of 22 deceased, freeze-thawed common brushtail possums at the University of Tasmania (Table 1). The possums originated from commercial hunter-killed stock, collected from mixed agricultural-woodlands on private properties in Northern Tasmania.

### DNA extraction

DNA extraction from swabs obtained from prospective sampling was performed using the QIAmp DNA mini kit (QIAGEN, Victoria, Australia). Swabs were vigorously shaken in TE (Tris-EDTA pH 8) buffer, followed by DNA extraction using the ‘DNA purification from tissues’ protocol as per the manufacturer’s instructions. DNA was stored at −20 °C until further use. DNA extractions performed on retrospective samples used the SIGMA Genelute bacterial DNA extraction kit in accordance with the manufacturer’s instructions.

### Order specific 16S *Chlamydiales* PCR

To screen marsupials for *Chlamydiales*, an 800 bp fragment of the 16S rRNA gene partially covering the *Chlamydiales* signature sequence was amplified. PCR reactions consisted of 25 µl volumes containing 1.5 µl of each primer and 4 µl of template DNA, products were purified and sequenced following previously described methods^13^.

### *Chlamydia pecorum* MLST PCR scheme

Samples with 16S rRNA sequences with a closest BLAST match to *Chlamydia pecorum* were subjected to *C. pecorum* MLST. The seven conserved *C. pecorum* specific housekeeping genes from the *C. pecorum* MLSA scheme was amplified following previously described methods^8^.

### Sequence analysis and phylogenetic parameters

16S rRNA and *gidA* PCR products was purified using the Roche High Pure PCR Product Purification Kit (Roche, New South Wales, Australia) following the manufacturer’s instructions. Purified PCR products were sequenced by Macrogen Inc. (Seoul, Korea). Chromatograms of forward and reverse sequences were aligned in the Geneious R9.1.3 software package^27^. A consensus sequence was derived from each alignment and trimmed to the maximum length possible, *gidA* consensus sequences were trimmed to 474bp^8^. 16S rRNA gene consensus sequences, GenBank representatives of the closest BLAST match for each of the *Chlamydiaceae*, *Ca*. Rhabdochlamydiaceae and *Ca*. Parilichlamydiaceae families and an appropriate outgroup were then aligned in Geneious R9.1.3 using the MUSCLE plugin^28^ with default parameters. The alignments comprised of 18, 7 and 7 16S rDNA sequences trimmed to a length of 588, 368 and 701 bp, respectfully. For *gidA*, 12 sequences including consensus sequences, representatives of the closest PubMLST match^16^ from all possible host species and an appropriate outgroup were aligned in Geneious R9.1.3 using the MUSCLE plugin with default parameters. Bayesian phylogenies were constructed in Geneious R9.1.3 using the MrBayes plugin^29^ under the HKY85 substitution model. Run parameters included four Markov chain Monte Carlo (MCMC) chains with a million generations, sampled every 3,000 generations, and with the first 100,000 trees discarded as ‘burn-in’.
